# HDAC inhibitor valproic acid protects heart function through Foxm1 pathway after acute myocardial infarction

**DOI:** 10.1016/j.ebiom.2018.12.003

**Published:** 2018-12-11

**Authors:** Shuo Tian, Ienglam Lei, Wenbin Gao, Liu Liu, Yijing Guo, Jeffery Creech, Todd J. Herron, Shaoxiang Xian, Peter X. Ma, Y. Eugene Chen, Yongqing Li, Hasan B. Alam, Zhong Wang

**Affiliations:** a Department of Cardiac Surgery, Cardiovascular Center, The University of Michigan, Ann Arbor, MI 48109, USA; b Faculty of Health Sciences, University of Macau, Avenida de Universidade, Taipa, Macau, PR China; c The First Affiliated Hospital, Guangzhou University of Chinese Medicine, Guangzhou 510405, PR China; d Department of Spine Surgery, Xiangya Spinal Surgery Center, Xiangya Hospital, Central South University, Changsha 410008, China; e Cardiovascular Center, Department of Internal Medicine, University of Michigan Medical Center, Ann Arbor, MI 48109, USA; f Department of Biologic and Materials Science, University of Michigan, Ann Arbor, MI 48109, USA; g Department of Surgery, University of Michigan Health System, Ann Arbor, MI 48109, USA

**Keywords:** Valproic acid, Myocardial infarction, Foxm1, Cardiomyocyte protection

## Abstract

**Background:**

Epigenetic histone acetylation is a major event controlling cell functions, such as metabolism, differentiation and repair. Here, we aim to determine whether Valproic acid (VPA), a FDA approved inhibitor of histone deacetylation for bipolar disease, could protect heart against myocardial infarction (MI) injury and elucidate key molecular pathways.

**Methods:**

VPA was administrated to MI rats at different time points, onset and after MI injury. Echocardiography, histology, serum biology assays, and gene expression, inhibition, and over-expression were performed to characterize the systolic function, infarct size, gene and signaling pathways.

**Findings:**

VPA treatment reduced the infarct size by ~50% and preserved the systolic function of heart after acute MI in rats. Even 60 min after infarction, VPA treatment significantly decreased infarct size. Furthermore, long-term treatment of VPA markedly improved myocardial performance. VPA regulated gene expression essential for cell survival and anti-inflammatory response. Consequently, oxidative stress and cell death were notably reduced after VPA treatment. Moreover, Foxm1 was identified as a potential key target of VPA. Overexpression of Foxm1 provided similar heart protective effect to VPA treatment. Particularly, both VPA treatment and Foxm1 over-expression repressed inflammatory response after MI for heart protection. In contrast, inhibition of Foxm1 activity abolished the cardiac protective effect of VPA. VPA mediated CM protection through Foxm1 upregulation was also identified in a human ESC derived CM hypoxia/reperfusion system.

**Interpretation:**

VPA treatments significantly reduce cardiac damage after MI and the cardioprotective effect of VPA is likely mediated via Foxm1 pathway.

**Fund:**

This work was mainly supported by 1R01HL109054.

**Research in context**

**Evidence before this study**

Epigenetic histone acetylation is a major event controlling cell functions, such as metabolism, differentiation and repair. In particular, cells under stress or injury are often accompanied with histone deacetylation. Therefore, maintaining proper histone acetylation could be key to treating heart disease.

**Added value of this study**

In this study, we tested the hypothesis that valproic acid (VPA), an FDA approved histone deacetylases (HDAC) inhibitor for bipolar, could protect heart against myocardial infarction (MI) injury in rats and elucidated key molecular pathways for heart protection. Our results showed that VPA treatment, even 60 min after infarction, significantly reduced the infarct size and preserved the systolic function of heart after acute MI in rats. Furthermore, long-term treatment of VPA markedly improved myocardial performance. Moreover, Foxm1 was identified to be a potential key target of VPA. Overexpression of Foxm1 provided similar heart protective effect to VPA treatment. In contrast, inhibition of Foxm1 activity abolished the cardiac protective effect of VPA. VPA mediated cardiomyocyte (CM) protection through Foxm1 upregulation was also identified in a human CM hypoxia/reperfusion system.

**Implications of all the available evidence**

VPA treatments significantly reduce cardiac damage after MI and the cardioprotective effect of VPA is likely mediated via Foxm1 pathway. VPA's status as an approved clinical used drug for treatment of neurological diseases has an added advantage of being readily repurposed for treating patients with acute MI.

Alt-text: Unlabelled Box

## 1 Introduction

Cardiovascular disease (CVD) is the leading cause of death in the world [1]. In particular, myocardial infarction (MI), commonly known as heart attack, results in permanent heart muscle damage or death, and is the number one killer of heart patients. A typical human heart attack causes the loss of approximately 1 billion cardiomyocytes (CMs). Existing treatments for heart attack are primarily pharmacological and device-based, and do not address the underlying problem of CM loss. These treatments cannot regenerate the myocardium and rescue the injured ventricle [2]. These statistics underscore the critical need for developing effective therapeutic strategies to preserve the pumping function after heart attack.

Cell plasticity or cell state changes, including dedifferentiation and maturation, are critical in disease pathogenesis and prevention, and are likely key to successful cardiomyocyte protection and repair. Cell fate/state changes are accompanied and largely defined by extensive chromatin modifications, such as acetylation, methylation, phosphorylation, and ATP-dependent chromatin remodeling. Therefore, epigenetic regulation may play a key role in cardiac protection and regeneration. Elevated histone acetylation is often accompanied by cell stage changes in response to various signals, particularly acetylation at the super enhancer regions. Acetylation of histones is regulated mainly by two groups of enzymes, histone acetyl transferases (HATs) to add and histone deacetylases (HDACs) to remove acetyl groups. Importantly, deacetylation appears to be a major event after various injuries [3,4], suggesting that the deacetylation activity could be targeted for heart injury.

HDACs remove the acetyl groups from the lysine residues and render chromatin condensed and transcriptionally silenced [[5], [6], [7]]. HDAC inhibitors prevent this action and rescue gene expression. Various HDAC inhibitors have thus been found beneficial in treating several diseases. For example, they have been used as mood stabilizers and anti-epileptics. Recent advances have led to their use in treating cancers, parasitic, and inflammatory diseases [[8], [9], [10], [11]]. Research in cardiovascular field indicates that HDAC inhibitors may be used to treat supraventricular arrhythmia, myocardial infarction, cardiac remodeling, hypertension, and fibrosis [5,[12], [13], [14]]. Valproic acid (VPA) is an HDAC inhibitor and has been approved for treating epilepsy and bipolar disorders [15,16]. Epidemiology studies on patients taking VPA suggest that VPA may have protecting effects on the cardiovascular system [17,18]. However, whether VPA improves cardiac function after MI and what is the underlying molecular mechanism have not been conducted.

In this study, we set out to examine the effects of HDACi VPA on cardiac protection. We demonstrated that administration of VPA, a selective class I HDAC inhibitor [19] and a FDA approved drug for epilepsy and bipolar disorders, to the ischemic heart greatly improve cardiac function. Specifically, we found that VPA reduced the infarct size by ~45% and enhanced cardiac function by ~50%. VPA significantly reversed the gene expression altered by MI related to cardiomyocyte metabolism and inflammation response. Furthermore, we identified that VPA treatment increased the expression of Foxm1, a potential master regulator of gene expression for heart protection and repair. Overexpression of Foxm1 also led to rescue cardiac function after MI. Both VPA treatment and Foxm1 over-expression repressed inflammatory response after MI for heart protection. Conversely, inhibition of Foxm1 activity abolished the cardiac protective effect of VPA. Our results thus identified a VPA-mediated Foxm1 pathway for cardiac protection involving cell survival and anti-inflammatory response after MI. Because VPA has already been approved as a clinical drug for treating bipolar disease and other neuron diseases, this study provided strong evidence that VPA could be an ideal drug readily repurposed for treating patients with acute heart attack.

## 2 Materials and methods

### 2.1 Animal experiment

All experiments were approved by the Animal Care and Use Committee of the University of Michigan and were performed in accordance with the recommendations of the American Association for the Accreditation of Laboratory Animal Care.

### 2.2 MI models and treatment

Myocardial ischemia/reperfusion operation was carried out in rats as described previously [20]. Briefly, rats were anaesthetized with ketamine (100 mg/kg) and xylazine (10 mg/kg). Myocardial ischemia was performed by occlusion of the left descending coronary artery (LAD) using 6–0 silk sutures. After 60 min of ischemia, the myocardium was then reperfused. For acute MI studies, rats were randomly divided into 4 groups: Sham, MI, MI + VPA and MI + VPA 60 min post dose. MI + VPA groups were injected intraperitoneally with VPA at the onset or at 60 min of LAD occlusion. The rats in Sham and MI groups were administered with a corresponding dose of the saline. Another dose was administered at 12 h after surgery. To test the effects of Foxm1 in VPA treatment, we tested if Foxm1 inhibitor thiostrepton (TST) could abolish the protective effects of VPA after MI. Rats were randomly divided into 4 groups: MI, MI + VPA, MI + TST, MI + VPA + TST. VPA was treated as above and 50 mg/kg Foxm1 inhibitor TST was injected intraperitoneally daily two days before MI surgery and VPA treatment in MI + TST and MI + VPA + TST groups. 24 h later, heart functions were analyzed. To test the long-term effects of VPA, rats were randomly divided into 3 groups: Sham, MI and MI + VPA. In MI + VPA group, continually administration of VPA (250 mg/kg, twice daily) after Day 1 treatment was performed for 28 days before heart functions were analyzed.

Similar to rat operation, mice were anaesthetized with ketamine (100 mg/kg) and xylazine (10 mg/kg). MI was carried out by permanent occlusion of the left descending coronary artery (LAD) using 8–0 silk sutures as described in our previous studies [21,22]. Mice were randomly divided into 2 groups: MI + AAV-Luciferase (Luc) and MI + AAV-Foxm1. Foxm1 cDNA was cloned into AAV9 vectors driven by cTnT promoter. AAV virus was packaged in HEK293 cells by transfecting AAV9-TnT-Foxm1/Luciferase, AAV2/9n and pAd∆F6 (UPenn vector core). AAV virus was harvested after 3 days of transfection and purified by iodixanol density gradient ultracentrifugation as previously described [23]. The titer of AAV was determined by qPCR. AAV-9 virus containing cTnT-Luciferase (Luc group) or cTnT-Foxm1 (Foxm1 group) was intravenously injected to mice 6 days before MI surgery with a dose of 1 × 10^12^ gc/mice. 28 days after MI, mice were euthanized for assay.

### 2.3 Echocardiography

Echocardiography was performed after surgery. Left ventricular internal diameter end diastole (LVIDd) and end systole (LVIDs) were measured perpendicularly to the long axis of the ventricle. Ejection fraction (EF) and fractional shortening (FS) were calculated according to LVIDd and LVIDs. All echocardiography measurements were performed by a single-blinded investigator.

### 2.4 Triphenyltetrazolium chloride (TTC) staining

The hearts were frozen rapidly and sliced into five 2 mm transverse sections. The sections were incubated at 37 °C with 1% TTC in phosphate buffer (pH 7.4) for 10 min, fixed in 10% formaldehyde solution, photographed and calculated using Image J software. The infarct size was expressed as a percentage of infarct volume versus left ventricle volume.

### 2.5 Determination of serum CK, serum LDH, tissue SOD and tissue MDA

Blood samples were collected at 24 h after reperfusion and plasma was isolated. The creatine kinase (CK, MAK116, Sigma) and lactate dehydrogenase (LDH, MAK066, Sigma) level in plasma were measured according to the manufacturer's instructions. Ventricles were crushed to a powder using liquid nitrogen and homogenized in saline with the weight/volume ratio of 1:10. After centrifuging for 10 min at 3500 rpm, the supernatants were withdrawn for SOD (S311–10, Dojindo Molecular Technologies) and MDA (MAK085, Sigma) measurement according to the manufacturer's instructions. Bradford protein assay was performed to determine the protein concentration.

### 2.6 Histology assay

Histological studies were performed as previously described [21]. Briefly, animals were sacrificed and the hearts were perfused with 20% KCl. After fixed with zinc fixative solution (BD Pharmingen) and dehydrated by alcohol, the samples were embedded by paraffin and sectioned into 5 μm slides. The sections were processed for immunostaining, including Masson's trichrome, immunofluorescence and TUNEL assay (in situ cell death detection kit, Roche). Images were captured by Aperio (Leica Biosystems, Buffalo Grove, IL, USA) and a confocal microscope (Nikon, Melville, NY, USA) and analyzed by Image J software. For TUNEL assay, 3 animals per group were randomly selected and at least 4 slides per heart tissue were randomly selected to evaluate the percentage of dead cells. For each slide, 8 randomly selected fields were chosen from the border zone of the ischemic areas. The TUNEL positive cells and DAPI positive cells were counted by two independent observers blinded to treatment group and the cell death index was calculated. After Masson's trichrome staining, the epicardial infarct ratio was measured by dividing epicardial infarct lengths by the normal epicardial length of left ventricle. Endocardial infarct ratio was similarly measured. Infarct size was calculated as [(epicardial infarct ratio + endocardial infarct ratio) /2] × 100% [24].

### 2.7 Western blot

Proteins were extracted in lysis buffer followed by centrifugation at 4^∘^C for 15 min at 12,000 rpm. Protein concentration was measured by Bradford protein assay and 40 μg of total protein was separated by SDS-PAGE and then transferred to polyvinylidene difluoride membranes. The membranes were blocked with 5% nonfat dry milk for 1 h at room temperature and then incubated with primary antibodies over night at 4 ^∘^C. After 3 washings with TBST, the membranes were incubated with secondary antibody in TBST solution for 1 h at room temperature. After 3 washings, the membranes were scanned and quantified by Odyssey CLx Imaging System (LI-COR Biosciences, USA).

### 2.8 RNA-seq

The LV tissue was collected and total RNAs were extracted by Trizol following manufacture's protocol. The total RNAs were treat with DNAse Turbo to remove genomic DNA. RNA quality was assessed using Agilent Bioanalyzer Nano RNA Chip. 1 μg of total RNA (RIN > 8) was used to prepare the sequencing library using NEBNext Stranded RNA Kit with mRNA selection module. The library was sequenced on illumina HiSeq 4000 (single end, 50 base pair) at the Sequencing Core of University of Michigan.

### 2.9 RNA-seq data analysis

RNA-seq data was quantified using Kallisto (Version 0.43.0) [25] with parameters: –single -b 100 -l 200 -s 20 using the Rnor6.0 (ensembl v91). The estimated transcript counts were exported by tximport [26] for Deseq2 [27] analysis. Differential expression was then calculated using Deseq2 default setting. Gene Ontology analysis was perform using David tools [28].

### 2.10 Human cardiomyocytes

Human embryonic stem cells (hESCs) were used to generate human CMs (hCMs) as previous publication [29]. Briefly, full confluent single layer hESCs were cultured in CDM3 medium to induce CM differentiation. CDM3 medium contained RPMI 1640 medium, 500 μg/ml O. sativa–derived recombinant human albumin, and 213 μg/ml l-ascorbic acid 2-phosphate. Medium was changed every 2 days. 6 μM GSK3 inhibitor CHIR99021 was supplemented during the first two days of differentiation, and 5 μM Wnt inhibitor IWR1 was supplemented during day 5 to day 6. Beating CMs were observed after day 10. At day 20, over 90% cTnT+ CMs were changed to RPMI1640 with no glucose and subjected in a hypoxia chamber saturated with 5% CO_2_/95% N_2_ to mimic ischemia condition. 6 h later, the CMs were replaced to CDM3 medium (normal glucose) with 5% CO_2_ at 37 °C for 3 h. 10 μM VPA dissolved in ddH2O was added 12 h before and during hypoxia/reperfusion (HR) treatment. Cells without HR treatment were used as control. Cells were collected for TUNEL, PCR and Western blot assays.

### 2.11 Statistical analysis

GraphPad Prism Software (version 7) was used for statistical analysis. Data are expressed as the mean ± SEM. Statistical comparisons between two groups were performed by Student's *t*-test, and more than two groups were performed by one-way ANOVA followed by post-hoc Turkey HSD analysis. Groups were considered significantly different at  P < .05.

## 3 Results

### 3.1 VPA treatment improved cardiac function and attenuated myocardial damage after acute MI

**Fig. 1:**
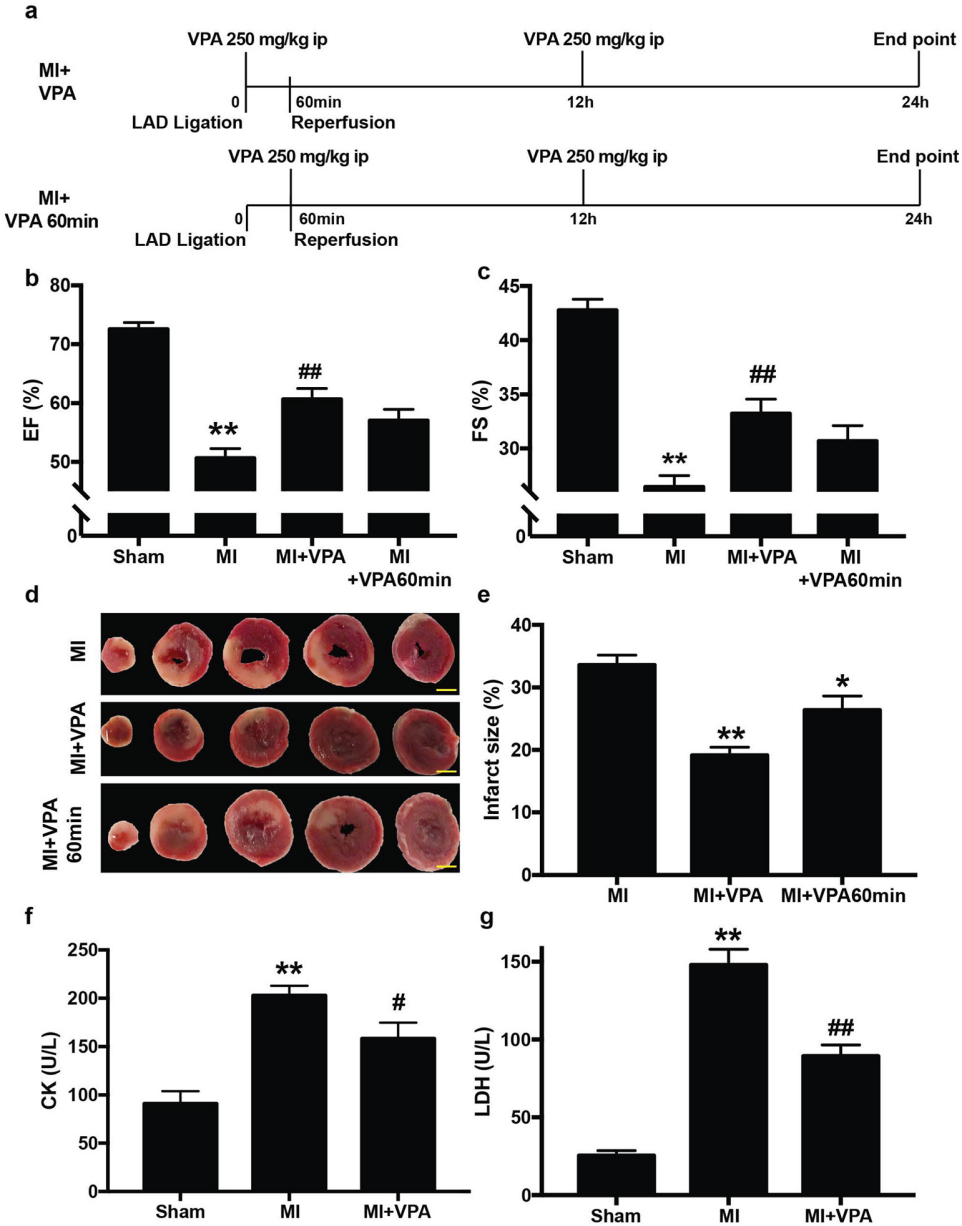
VPA treatment improved cardiac function and attenuated myocardial damage in rats at 24 h after MI. (a) Timeline of MI model generation and VPA treatment. (b) and (c) Echocardiographic measurements of ejection fraction (EF) and fractional shortening (FS) of Sham, MI, MI + VPA and MI + VPA 60 min rats at 24 h after MI. **, P < .01 vs Sham group; ##, P < .01 vs MI group; *n* = 9 except for MI + VPA 60 min group, which is 4. (d) Representative images of heart sections by TTC staining. Scale bar: 2.5 mm (e) Quantitative analysis of infarct size expressed as percentage of left ventricle. **, P < .01, *n* = 6. (f) Serum CK and (g) serum LDH levels. **, P < .01 vs Sham group; #, P < .05 vs MI group; ##, P < .01 vs MI group; *n* = 9. All samples were analyzed by one way ANOVA followed by post-hoc Turkey HSD analysis. Data were expressed as mean ± SEM.

To investigate the role of VPA on heart protection against MI injury, we generated MI rats by ischemia/reperfusion (ischemia for 1 h and reperfusion for 24 h) followed by administration of VPA to the animals. VPA was intraperitoneally injected at 250 mg/kg immediately and 12 h after coronary ligation (Fig. 1a). Echocardiography was performed to evaluate the cardiac function 24 h after MI. Left ventricular internal diameter end diastole (LVIDd) and end systole (LVIDs) were measured perpendicularly to the long axis of the ventricle. Ejection Fraction (EF) and fractional shortening (FS) were calculated according to LVIDd and LVIDs. Acute MI rats typically showed 52 ± 7% of EF and 28 ± 4% of FS, whereas VPA treatment improved the EF to 61 ± 6% and FS to 33 ± 4% (Fig. 1b, c). These data indicated that VPA treatment within 12 h of MI injury markedly improved cardiac function by rescuing roughly 50% of the functional loss caused by acute MI injury.

We next examined whether the improved cardiac function is associated with reduced myocardium death. The infarct size was examined 24 h after MI by triphenyltetrazolium chloride (TTC) staining. The infarct size was expressed as a percentage of infarct volume versus left ventricle volume. In MI only rats, 33.59 ± 3.54% infarction of the left ventricle was induced 24 h post coronary ligation in rats. Notably, VPA treatment significantly reduced the infarction size to 19.14 ± 2.89% of the left ventricle compared to saline controls (Fig. 1d, e). Strikingly, two shots of VPA within 24 h of MI injury significantly reduced the damaged area by around 45% after myocardial infarction. Furthermore, in a more clinical relevant setting, when VPA was administrated 60 min after coronary ligation, VPA treated rats showed significantly reduced infarct size of 26.34 ± 1.95% and improved cardiac function with a EF of 57 ± 2% and FS of, 31 ± 1% (Fig. 1a-e).

Myocardial cell death occurs several hours after occlusion of the coronary artery. As myocardial damage indicators, creatine kinase (CK) and lactate dehydrogenase (LDH) expression levels are dramatically elevated after MI and reach peaks at around 24 h [30]. We therefore measured the plasma LDH and CK levels at 24 h after MI to determine the effects of VPA on reduction of MI injury. In the acute MI rats without VPA treatment, high levels of LDH leakage (147.92 ± 8.98 U/l) and CK leakage (202.88 ± 30.63 U/l) were observed (Fig. 1f, g). In contrast, VPA treatment significantly reduced the MI-induced release of LDH (89.31 ± 6.42 U/l) and CK (158.31 ± 43.53 U/l). Thus, compared with MI rats, administration of VPA decreased myocardial damage after MI. Together, our results indicated that VPA had a significant role in cardiac protection after MI injury.

### 3.2 VPA treatment decreased oxidative stress and cell apoptosis after acute MI

**Fig. 2:**
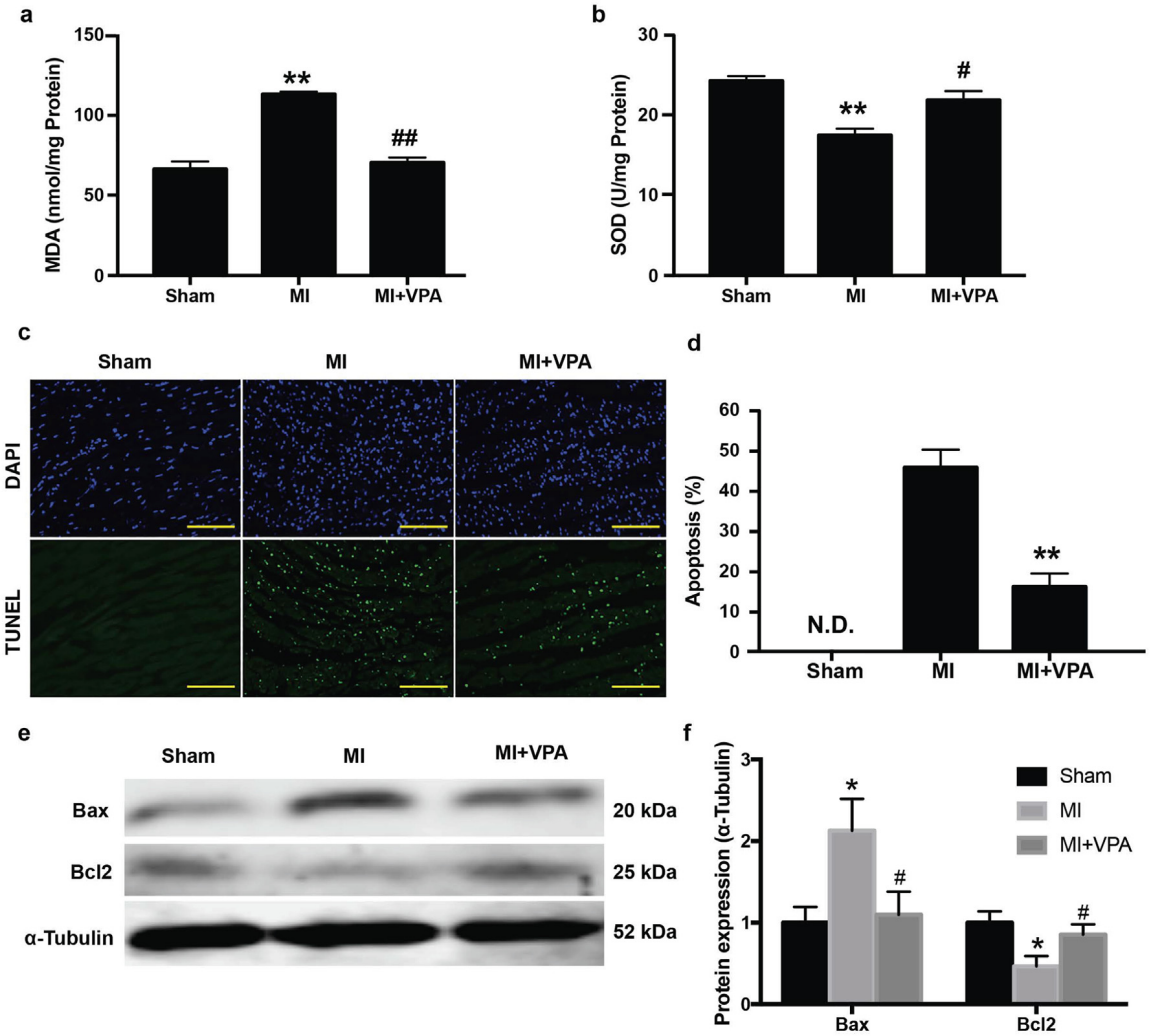
VPA treatment decreased oxidative stress and cell apoptosis after acute MI. Myocardial MDA (a) and SOD (b) activities. **, P < .01 vs Sham group; #, P < .05 vs MI group; ##, P < .01 vs MI group; n = 6. (c) Representative images of in situ detection of apoptotic cells by TUNEL staining. Myocardial tissues were stained and analyzed with TUNEL (green) and counterstained with DAPI (blue). Scale bar: 200 μm (d) Quantitative analysis of TUNEL positive cells expressed as percentage of DAPI stained cells. **, P < .01, *n* = 3, N.D., No Detectable. (e) Representative images of Western blot of Bax and Bcl2 expression from Sham, MI and MI + VPA treated rats. (f) Relative intensity of Bax/Bcl2 over α-Tubulin. *, P < .05 vs Sham group; #, P < .05 vs MI group, *n* = 3. All samples were analyzed by one way ANOVA followed by post-hoc Turkey HSD analysis. Data were expressed as mean ± SEM.

Oxidative stress induced CM apoptosis is the major cause of cell death after MI. To investigate the beneficial effects of VPA treatment, oxidative stress level and cell apoptosis were measured after MI with and without VPA. Antioxidant enzyme activities in the left ventricles were measured in different groups 24 h after MI. MI significantly increased the oxidative stress as evidenced by increased MDA activities (112.95 ± 1.49 nmol/mg protein vs 66.27 ± 4.75 nmol/mg protein in Sham group) and decreased SOD activities (17.44 ± 0.80 U/mg protein vs 24.25 ± 0.57 U/mg protein in Sham group) (Fig. 2a,b). However, administration of VPA significantly reduced the MI induced oxidative stress as measured by normalized activities of MDA (70.36 ± 3.16 nmol/mg protein) and SOD (21.85 ± 1.10 U/mg protein) (Fig. 2a,b), suggesting that VPA treatment notably attenuated MI induced oxidative stress. Oxidative stress is known to induce cell apoptosis. To study the effect of VPA on apoptosis, we measured the cell apoptosis in the border zone by TUNEL staining. Compared to the control group, the TUNEL-positive nuclei were dramatically increased in MI group. VPA treatment resulted in a pronounced reduction of TUNEL-positive cells, suggesting that VPA prevented cell apoptosis and enhanced cell survival after MI (Fig. 2c,d). Expression of apoptotic related protein Bax and Bcl2 was further investigated [31]. Compared with Sham control group, MI significantly increased Bax expression and decreased Bcl2 expression. VPA treatment reversed abnormal Bax and Bcl2 expression (Fig. 2e,f). These results indicated that administration of VPA after MI led to reduction of oxidative stress and cell apoptosis and improvement of myocardial outcome after MI.

### 3.3 VPA treatment improved long-term myocardial performance after MI

**Fig. 3:**
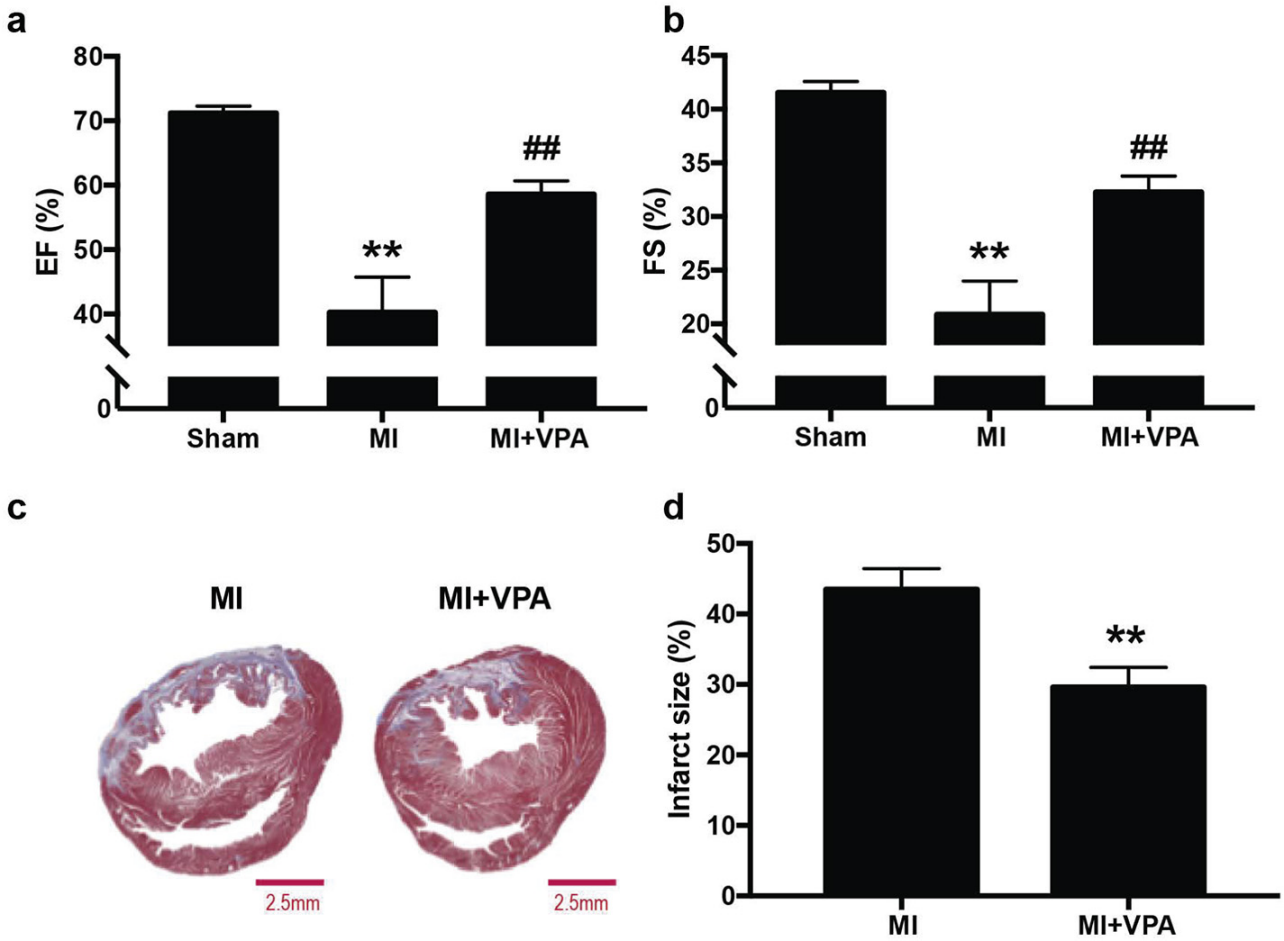
Long-term treatment of VPA restored cardiac function and scar size at 28 day after MI in rats. (a) and (b), Echocardiographic measurements of EF and FS of Sham, MI and MI + VPA rats at 28 days after MI. **, P < .01 vs Sham group; ##, P < .01 vs MI group; n = 9. (c) Representative images of heart sections by Masson's Trichrome staining. Scale bar: 2.5 mm (d) Quantitative analysis of infarct size expressed as percentage of left ventricle. **, P < .01, n = 6. All samples were analyzed by one way ANOVA followed by post-hoc Turkey HSD analysis. Data were expressed as mean ± SEM.

To determine the long-term effects of VPA treatment in improving myocardial performance, daily administration of VPA was performed after MI. 28 days after MI, a typical significant reduction of cardiac function was observed in MI group compared to sham group and the EF and FS values were 40 ± 5% and 21 ± 3%, respectively. VPA treatment resulted in 59 ± 2% of EF and 32 ± 1% of FS (Fig. 3a, b). The histological assay further indicated that VPA treatment significantly improved long-term myocardial performance by reducing the infarction size to 29.62 ± 2.80% compared with MI group (43.50 ± 2.96%) (Fig. 3c, d). The results indicated that continually administration of VPA prevented infarction progress and rescued cardiac function after MI.

### 3.4 VPA treatment reverses the expression of a variety of gene categories for heart protection

**Fig. 4:**
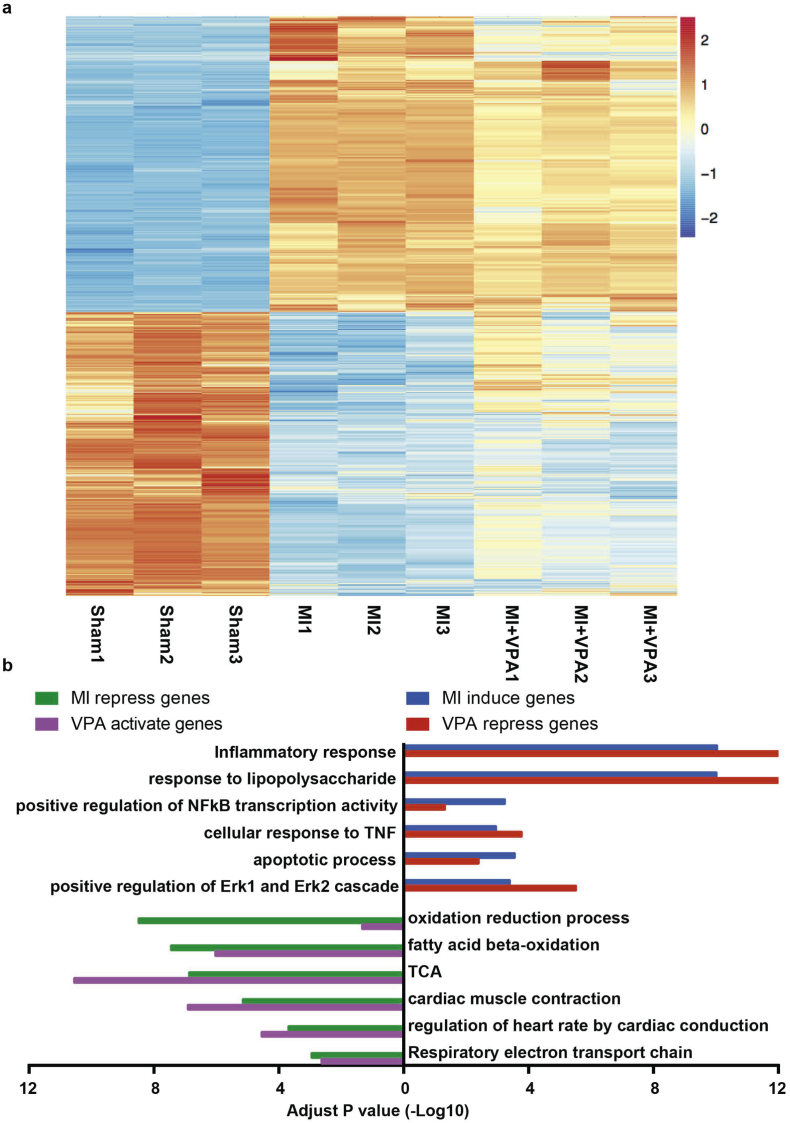
VPA treatment restored the gene expression change after MI. (a) Heatmap of genes differentially expressed in MI versus Sham. Scale is row z score. Differential genes are identified as ≥2-fold change and false discovery rate FDR < 0.05. Upper half of heatmap is genes upregulated in MI and repressed by VPA. Lower half is the cluster of genes downregulated by MI and reversed by VPA. (b) Gene ontology of four group of genes: MI induced (versus Sham), VPA repressed (versus MI), MI repressed (versus Sham) and VPA activated (versus MI) genes. x axis is the -log_10_(*p*-value).

To investigate the gene regulation mechanism mediated by VPA in reducing myocardial MI injury, we performed genome-wide transcription profile from LV tissue using RNA-seq in sham and MI rats with and without VPA treatment. Compared to sham hearts, MI hearts showed 961 upregulated genes and 1177 downregulated genes (abs(LogFC) > 1, FDR < 0.05). Moreover, treatment of VPA significantly reversed the gene expression alternations induced by MI (Fig. 4a and Fig. S1). Gene ontology analysis showed that VPA suppressed genes included those involved in cell death and pro-inflammation, while VPA promoted genes included those involved in oxidation reduction and metabolism (Fig. 4b) [32,33]. In addition, VPA treatment reduced the cell death after LPS induced cell death (Fig. S2). These results indicated that VPA treatment systematically attenuated MI induced myocardial injury.

### 3.5 Foxm1 is likely a key target of VPA for MI protection

**Fig. 5:**
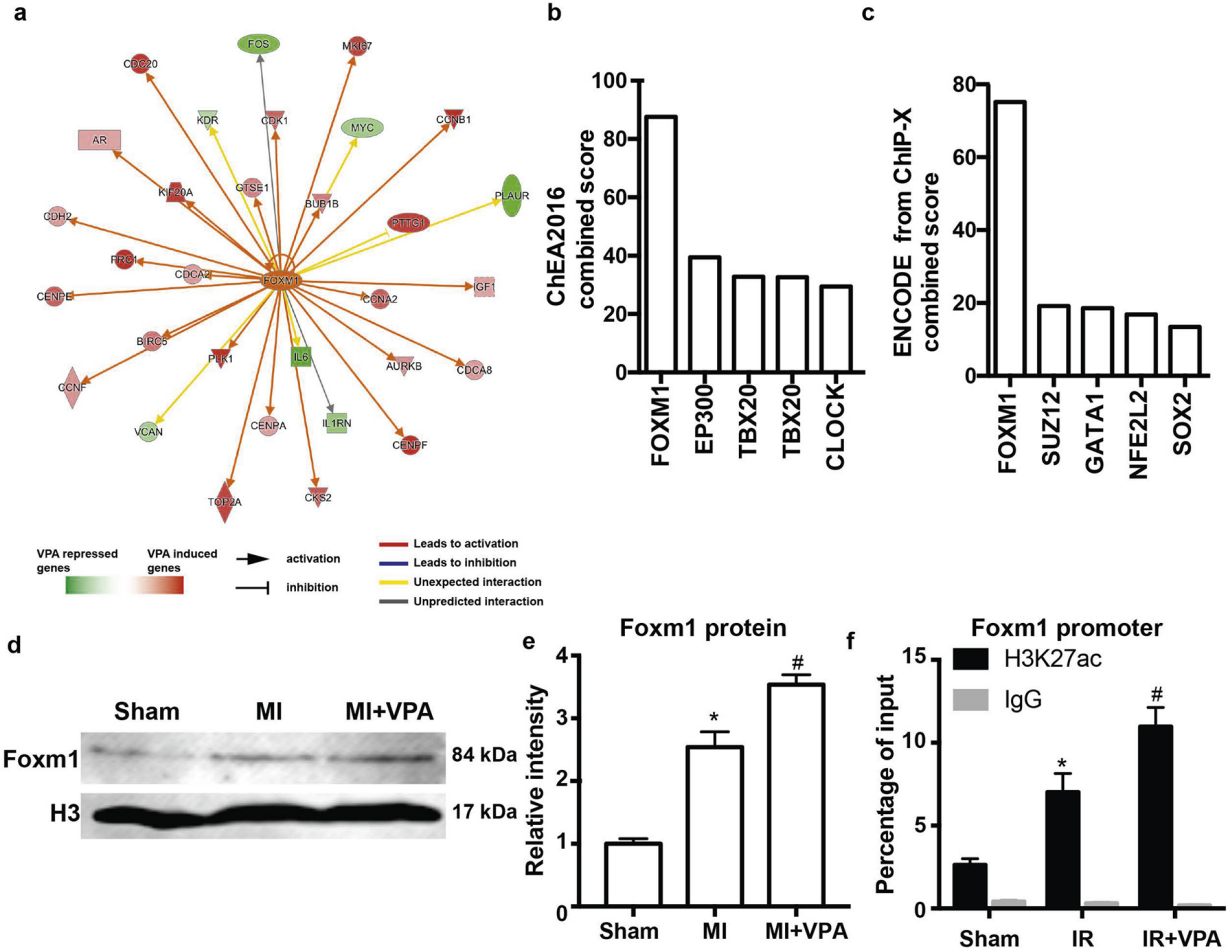
Foxm1 was a potential key target of VPA for MI protection. (a) Overlap of VPA and Foxm1 target genes by Ingenuity Pathway Analysis (P < 1.0 × 10–24, activation z score = 3.450). (b) and (c) Overlap of gene sets in public database with VPA target genes using Enrichr analysis. (d) Western blot of heart tissue from Sham, MI and MI + VPA treated rats. (e) Relative intensity of Foxm1 over histone H3 from 5 replicates of western blot. *, P < .05 vs Sham group, #, P < .05 vs MI groups, *n* = 5. (f) ChIP using H3K27ac antibody at Foxm1 promoters. *, P < .05 vs Sham group, #, P < .05 vs MI groups, n = 5. All samples were analyzed by one way ANOVA followed by post-hoc Turkey HSD analysis. Data were expressed as mean ± SEM.

To identify potential downstream regulators underlying the therapeutic mechanisms of VPA treatment during MI, we examined VPA regulated genes by applying Ingenuity Pathway Analysis [34]. Through the upstream regulator analysis, we found that Foxm1 was a top candidate transcription factor to regulate VPA target genes (Fig. 5a). We also applied Enrichr [35] to independently identify gene sets in the public database that overlapped with VPA target genes after MI in our studies. Foxm1 ChIP-seq in U2OS cells and Foxm1-encode has the highest overlap with the VPA regulated genes in the CHEA2016 and Encode database (Fig. 5b, c). Importantly, Foxm1 was 4-fold higher in VPA treated MI heart compare to MI hearts. Foxm1 protein level was elevated after MI and further increased after VPA treatment (Fig. 5d, e). We also detected that H3K27ac modification at Foxm1 promoter was significantly higher in VPA treated hearts (Table S1, Fig. 5f), suggesting that VPA could upregulate Foxm1 expression by altering the histone acetylation status of Foxm1. These results strongly suggested that Foxm1 is a downstream regulator of VPA in preventing MI injury. Judging from the potential role of Foxm1 in cardiomyocyte growth [36], it is highly likely that Foxm1 is a major mediator of VPA to potentiate a feed-forward regulatory circuitry to open the chromatin for specific gene activation and protect CMs from ischemia.

### 3.6 Foxm1 overexpression improved cardiac function and reduced infarction after MI

**Fig. 6:**
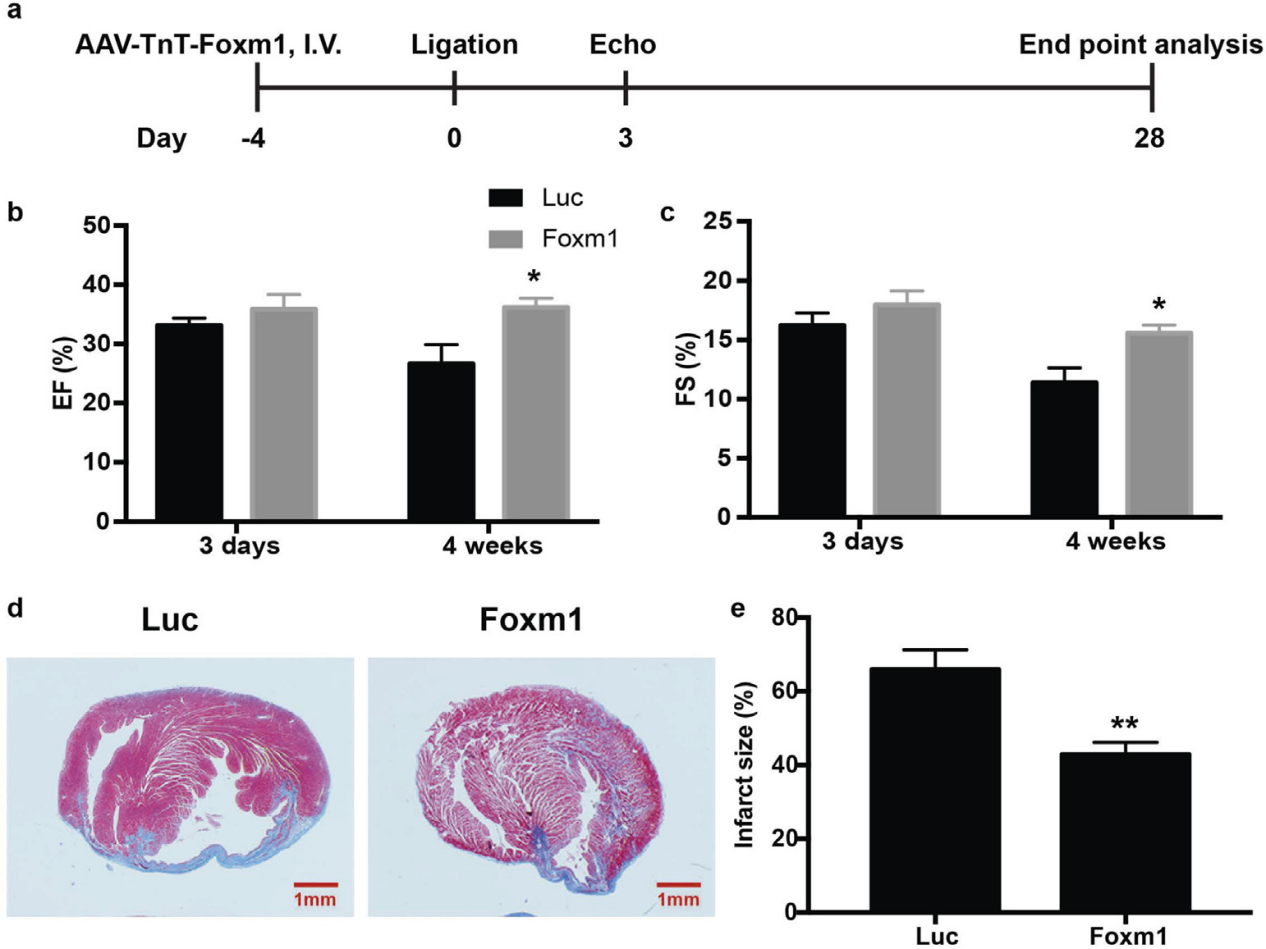
Foxm1 overexpression improved cardiac function and reduced infarction after MI. (a) Experimental design. (b) and (c) Echocardiographic measurements of EF and FS of MI + Luc and MI + Foxm1 mice at 3 days and 28 days after MI. *, P < .05, n = 6. (d) Representative heart cross sections stained with Masson's trichrome staining. Scale bar: 1 mm. (e) Quantitative analysis of infarct size expressed as percentage of left ventricle. **, P < .01, n = 6. All samples were analyzed by two tailed unpaired student's *t*-test. Data were expressed as mean ± SEM.

To determine if Foxm1 is a key regulator of VPA in cardiac protection after MI injury, we investigated the effect of Foxm1 overexpression after MI in mouse (Fig. 6a). We used AAV9-TnT system to induce cardiac specific overexpression of Foxm1 [37]. The AAV9 viruses containing either Luciferase (Luc group) or Foxm1 were delivered by I.V. injection. Detection of Luciferase by IVIS imaging confirmed the specific delivery and expression of the viruses to heart (Fig. S3a). Foxm1 overexpression was also confirmed by RT-qPCR (Fig. S3b) and Western blot (Fig. S3c, d). Echocardiography analysis was performed at day 3 and day 28 after MI. MI mice in Luc group showed 33 ± 1% of EF and 16 ± 1% of FS on day 3, whereas Foxm1 overexpression improved the EF to 36 ± 3% and FS to 18 ± 1%. The Luc group showed an average EF of 27 ± 3% and FS of 11 ± 1% 28 days after MI, while Foxm1 overexpression significantly prevented the infarction progress and maintained the cardiac function with an average EF of 36 ± 2% and FS of 16 ± 1% (Fig. 6b, c). Consisted with the echocardiography data, the histology assay showed significant reduction of infarction size in Foxm1 overexpression group (65.90 ± 5.33%) compared with Luc group (42.92 ± 3.23%) at day 28 after MI (Fig. 6d, e). Collectively, these data indicated that Foxm1 overexpression not only attenuated acute myocardial infarction injury, but also prevented ventricle remodeling and infarction expansion after MI.

### 3.7 Inhibiting Foxm1 activity abolished the heart protective role of VPA after MI

**Fig. 7:**
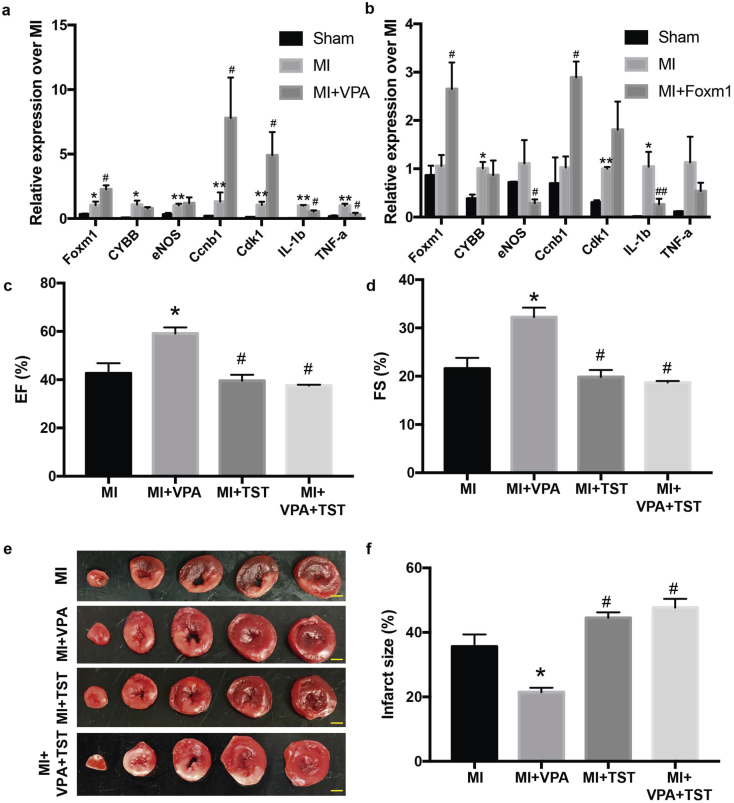
Inhibiting Foxm1 activity abolished the heart protective role of VPA after MI. (a) qPCR validation of genes regulated by VPA treatment after MI in rats. (b) qPCR analysis of VPA regulated genes in Foxm1 overexpression MI mice. *, P < .05 vs Sham group; **, P < .01 vs Sham group; #, P < .05 vs MI group; ##, P < .01 vs MI group; n = 3. (c) and (d) Echocardiographic measurements of ejection fraction (EF) and fractional shortening (FS) of Sham, MI, MI + VPA, MI + TST and MI + VPA + TST rats at 24 h after MI. *, P < .05 vs MI group; #, P < .05 vs MI + VPA group; n = 3. (e) Representative images of heart sections by TTC staining. Scale bar: 2.5 mm (f) Quantitative analysis of infarct size expressed as percentage of left ventricle. *, P < .05 vs MI group; #, P < .05 vs MI + VPA group; n = 3. All samples were analyzed by one way ANOVA followed by post-hoc Turkey HSD analysis. Data were expressed as mean ± SEM.

To test whether Foxm1 is a key downstream target of VPA for cardiac protection, we first examined the expression of several targets we identified in VPA treated MI rats. We identified that VPA treatment led to increased expression of proliferation genes Ccnb1 and Cdk1 as well as decreased expression of TNF and IL-1b after MI (Table S1, Fig. 7a). Meanwhile, Foxm1 overexpression in MI mice also increased the expression of Ccnb1 and Cdk1 and reduced expression of TNF and IL-1b expression (Table S1, Fig. 7b). These data suggested that both VPA and Foxm1 regulated common downstream targets to protected MI induced CM death by anti-inflammation and promotion of cell proliferation after MI. We then tested whether Foxm1 was required for the protective role of VPA in MI rats. We applied thiostrepton (TST), a known specific Foxm1 inhibitor [38,39], in MI rats treated with VPA at the concentration of 50 mg/kg. Treatment of TST did not change the cardiac function and infarction area in the MI rat (Fig. 7c-f). Compared to VPA treated MI rats, rats with VPA and TST did not show functional recovery (Fig. 7c, d). The effect of VPA protection in reducing the infarct size was also abolished after TST treatment (Fig. 7e, f). Altogether, our results revealed that Foxm1 pathway is a key pathway mediated by VPA for cardiomyocyte protection from MI injury.

### 3.8 VPA protected against hypoxia/ischemia (HR)-induced apoptosis in human CMs (hCMs) with upregulation of Foxm1

**Fig. 8:**
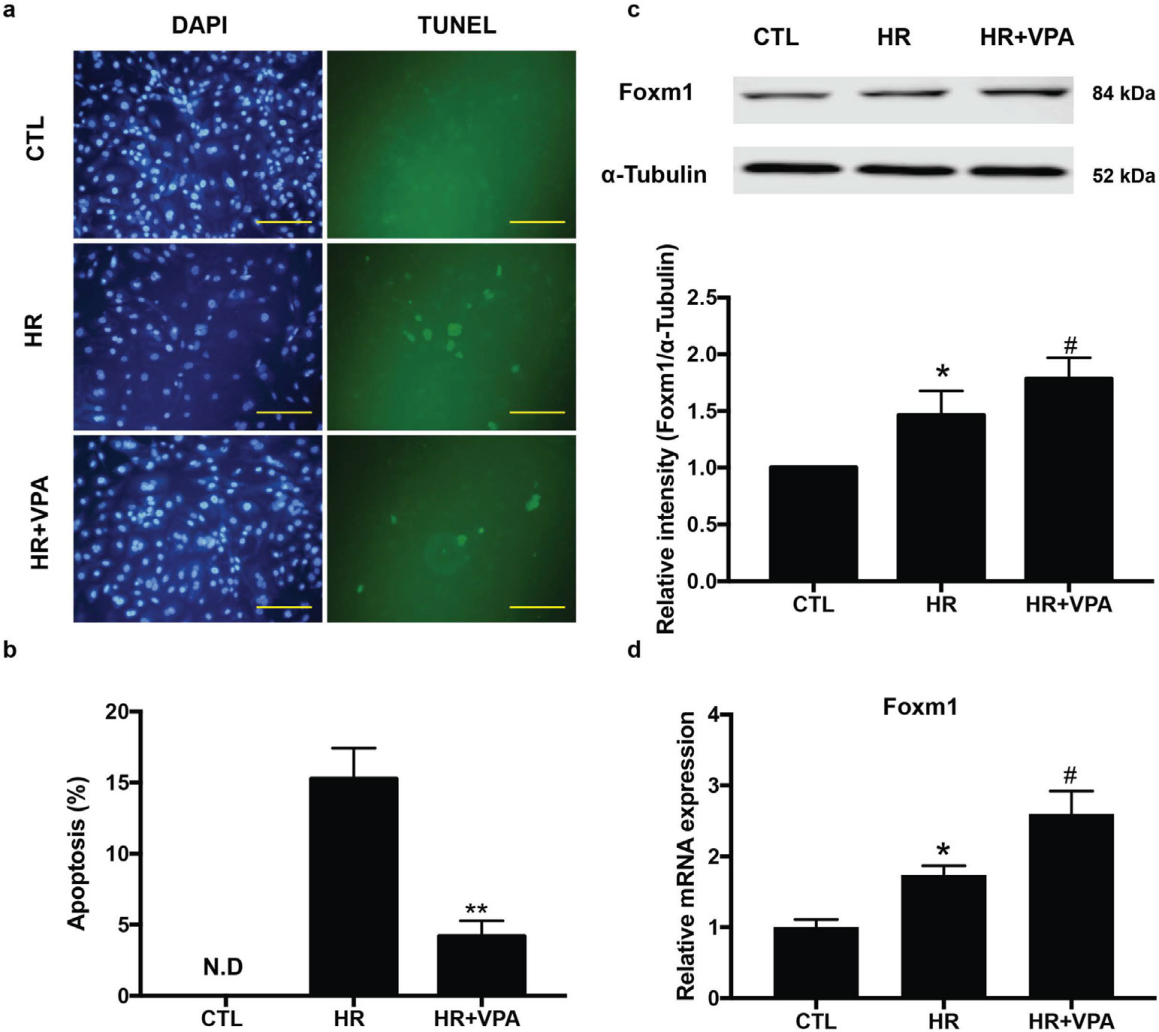
VPA treatment protected human CM apoptosis by upregulation of Foxm1. (a) Representative images of in situ detection of apoptotic cells by TUNEL staining. Apoptotic cells were stained and analyzed with TUNEL (green) and counterstained with DAPI (blue). Scale bar: 100 μm (b) Quantitative analysis of TUNEL positive cells expressed as percentage of DAPI stained cells. **, P < .01, n = 3 (Student's t-test), N.D., No Detectable. (c) Western blot and relative intensity of Foxm1 in human CMs treated with normal Control (CTL), HR and HR + VPA. *, P < .05 vs CTL group, #, P < .05 vs MI groups n = 6. (d) qPCR analysis of Foxm1 expression after HR and HR + VPA treatment. *, P < .05 vs CTL group; #, P < .05 vs HR group; n = 3. All samples were analyzed by one way ANOVA followed by post-hoc Turkey HSD analysis. Data were expressed as mean ± SEM.

To determine if VPA-induced Foxm1 pathway also protects hCM functions under MI conditions, we tested the effects of VPA on hCM apoptosis after MI using human embryonic stem cells derived cardiomyocytes (hESC-CMs). The hESC-CMs were subjected to 6 h hypoxia and 3 h reperfusion treatment with or without VPA treatment. Compared to the control group, substantial amount of TUNEL-positive nuclei were shown in the HR group. VPA treatment resulted in a pronounced reduction of TUNEL-positive cells, suggesting that VPA prevented hCM apoptosis and enhanced cell survival after MI (Fig. 8a, b). Consistent with the foxm1 activation in rodent model, we found that Foxm1 expression was significantly increased after VPA treatment in hCMs (Fig. 8c, d). Together with the in vivo data in rodent model, it is likely that VPA treatment led to reduction of HR-induced CM apoptosis by upregulation of Foxm1 expression. Furthermore, these hCM results provided solid evidence for clinical studies of repurposing VPA in effectively treating acute MI patients.

## 4 Discussion

Cardiomyocyte loss is the fundamental challenge for heart functional recovery after an ischemic heart attack. In this study, we show that Class I HDAC inhibitor VPA exerts prominent cardio-protective role after MI, which is characterized by significant reduction of infarct size, cell death, circulation markers, and cardiac remodeling with enhanced cardiac function. We show that VPA treatment increases the cardiomyocyte metabolic expression program after MI and suppresses the inflammation response. Furthermore, VPA treatment activates a Foxm1-mediated feed-forward gene expression pathway, which plays an important role in cell survival after MI.

In this study we demonstrate that VPA could efficiently treat MI heart injury as indicated by reduced infarcted area and improved cardiac function. Since VPA is an HDAC inhibitor that promotes histone and protein acetylation, these results suggesting that modulating the epigenetic response could effectively protect heart from MI injury. We find that HDAC activities are upregulated and histone acetylation decreased in H9C2 cells after 4 h hypoxia and 2 h reperfusion treatment, whereas VPA treatment significantly reverse HR induced HDAC activity and restore the loss of histone acetylation, suggesting that VPA protects the cardiomyocyte under injury through HDAC inhibition (Fig. S4). Consistent with our results, other reports show that HDAC inhibitors such as TSA and SAHA reduce the infarct size after MI [14,40]. Moreover, when we compare the cardiac protective effect of VPA and SAHA at the setting of MI, VPA shows better preservation of cardiomyocyte preservation after MI (Fig. S5a, b). We propose that inhibition of HDAC 1–3 is most efficient in heart protection. VPA specifically inhibits HDAC 1–3, while TSA and SAHA inhibit a boarder HDACs [[1], [2], [3], [4], [5], [6], [7], [8], [9], [10]] [41]. In agreement with this, a published study showing that HDAC6 (class IIb) inhibitor Tubastatin A does not exhibit cardiac protective effects [42]. Therefore, the high efficiency of VPA in cardiac protection after MI injury could be due to its specificity of HDAC inhibition.

In addition to the efficient treatment of VPA in MI hearts, VPA also shows low toxicity and high safety in patients. According to latest WHO data, VPA is included in the WHO's list of Essential medicines [43], a list of medications considered to be most effective and safe to meet the most important needs in a health system. VPA has already been approved as a clinical drug for treating bipolar and other neuron disease with rarely occurring toxicity [44]. Although VPA has also shown some side effects, such as asthenia, drowsiness, nausea, tremor and vomiting, these effects have been well controlled in clinic during the 50-year history of VPA application. In one of our studies, we have treated the MI rats with continual VPA administration for 4 weeks, and observed a long-term beneficial effect of VPA in heart function without noticeable side effects. In addition, VPA has also been safely used to treat other ischemia diseases, such as brain injury and hemorrhagic shock in pig models [45,46]. Thus, VPA could be repurposed as an excellent drug for both acute infarction and subsequent long-term cardiac remodeling therapy.

Our studies indicate that the protective role of VPA in heart function after MI can be attributed to a number of cell activities that VPA regulates. In this study, we identify that VPA reduces the activation of TNF, and further, VPA treated rats show significant reduction in recruitment of inflammation cells at 3 days after MI (Fig. S2a). VPA also suppresses gene sets involved in TNF signaling (P < 1 × 10^−24^; Fig. S2b). Moreover, our results show that VPA could reduce the cell death after LPS induction (Fig. S2c). Thus, one function of VPA is to suppress the immune response to reduce the cell death in the boarder zone. Our results are consistent with a previous brain injury study showing that VPA inhibits the TNF-NF-κB activity and modulates immune response [47]. Other functions of VPA include anti-apoptotic and improved energy metabolism, which are also consistent with previous studies in hemorrhagic [48].

Importantly, by analyzing potential targets of VPA regulated genes, we identified Foxm1, a key factor involved in protecting against MI injury as a down-stream target of VPA. Previous studies have identified Foxm1 in regulating the neonatal cardiomyocyte proliferation [36]. Foxm1 also promotes lung regeneration after injury by repressing the inflammation response [49]. We have detected that treatment with VPA leads to increased expression of Foxm1. We have further demonstrated that overexpression of Foxm1 reduces the infarct size and preserves cardiac function after MI. Moreover, we have identified that overexpression of Foxm1 and VPA treatment lead to regulated expression of many common target genes, such as genes involved in the suppression of immune response and proliferation genes. Furthermore, we have discovered that inhibition of Foxm1 activity by a chemical inhibitor attenuates the cardio-protective effect of VPA after MI. VPA mediated Foxm1 pathway in CM protection has been further detected in an in vitro human CM HR system. Altogether, our results indicate that suppression of immune response, anti-apoptosis, and improvement of energy metabolism after VPA treatments through Foxm1 are likely the major mechanisms of cardio-protection exerted by VPA.

In summary, our study demonstrates that HDAC inhibitor VPA plays a significant cardiac protection role after MI. Since VPA has already been approved as a clinical drug for treating bipolar and other neuron diseases and has less severe side effects than other HDAC inhibitors currently used in heart injury studies, we are optimistic that following more relevant preclinical studies, VPA can be readily repurposed for treating patients with acute heart attack. Since HDAC inhibitors generally promote histone and protein acetylation and given the essential and broad role of the epigenetic modification in cell activities, such as cell metabolism, self-renewal, differentiation, and maturation, identifying novel targets in stimulating histone and protein acetylation could hold great promise in treating infarcted hearts and other organ injuries.

## Appendix A Supplementary data

Fig. S1. Volcano plot showing the effect of VPA on MI induced and repressed gene expression. Fig. S2. VPA inhibited immune response. (a) Immunostaining using F4/80, CD11b and Ly6c antibody in rats with or without VPA treatment 3 days after MI. scale bar: 25μm (b) VPA inhibited TNF network identified by Ingenuity Pathway Analysis (P < 1.0 × 10^−24^, activation z score = -4.066). (c). Treatment of VPA to neonatal rat myocytes reduced the LPS induced cell death. Fig. S3. Overexpression of Foxm1 at day 6 after injection of AAV9-TnT-Foxm1. (a) Bioluminescent image. Detection of Luciferase using AAV9-TnT virus after 6 days of virus delivery. (b) qPCR of Foxm1 expression after 6 days of injection of AAV9-TnT-Foxm1. **, P < 0.01 vs control, n = 3. (c) Representative images of Western blot of Foxm1 expression. (d) Relative intensity of Foxm1 over histone H3 from 3 replicates. *, P < 0.05 vs AAV9-TnT-Luc group, n = 3. All samples were analyzed by two tailed unpaired student’s t test. Data were expressed as mean ± SEM. Fig. S4. HDAC inhibition effects of VPA in H9C2 cells after HR. (a) HDAC activity of normal control (Ctl), HR and HR+VPA treated H9C2 cells. *, P < 0.05 vs Ctl group; #, P < 0.05 vs HR group, n = 3. (b) Representative images of western blot from control (Ctl), HR and HR+VPA treated H9C2 cells and relative intensity of histone H3 acetylation (AcH3) over histone H3 from 3 replicates. **, P < 0.01 vs Ctl group; #, P < 0.05 vs HR group, n = 3. All samples were analyzed by one way ANOVA followed by post-hoc Turkey HSD analysis. Data were expressed as mean ± SEM. Fig. S5. Effects of SAHA (125 mg/kg, SQ) on reducing infarct size at 24 h after MI in rats. (a) Representative images of heart sections by TTC staining. Scale bar: 2.5 mm (b) Quantitative analysis of infarct size expressed as percentage of left ventricle, n = 5. Data were expressed as mean ± SEM. Image 1

Supplementary material Image 2
