# Supplementary material for: HDAC inhibitor valproic acid protects heart function through Foxm1 pathway after acute myocardial infarction
Source: eBioMedicine. 2018 Dec 11;39:83–94. doi: 10.1016/j.ebiom.2018.12.003 (PMC6354709; doi:10.1016/j.ebiom.2018.12.003)

Figure S1

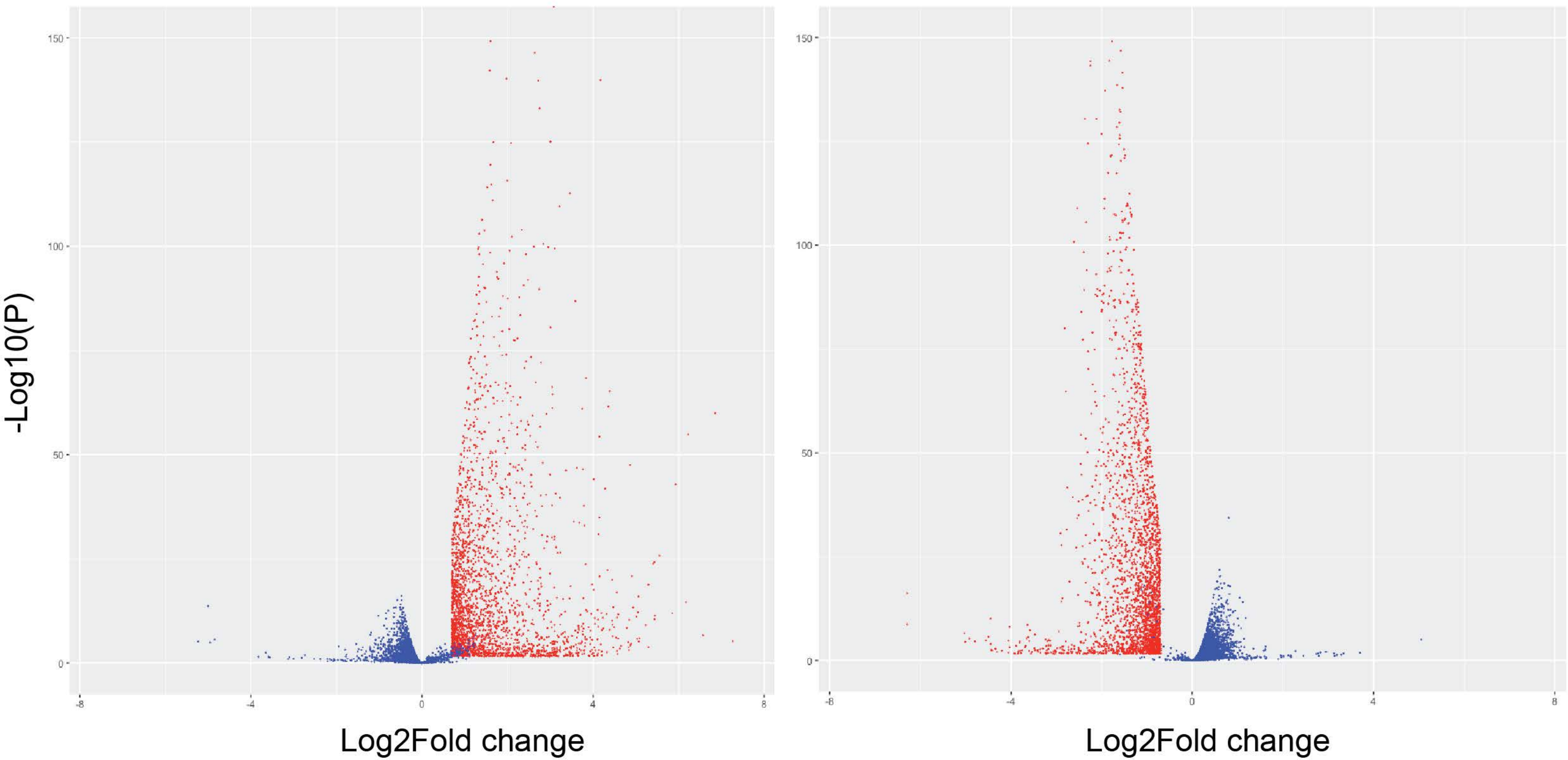

MI induced gene in red
VPA effect on all MI induced genes

MI repressedd gene in red
VPA effect on all MI repressed genes

Figure S2

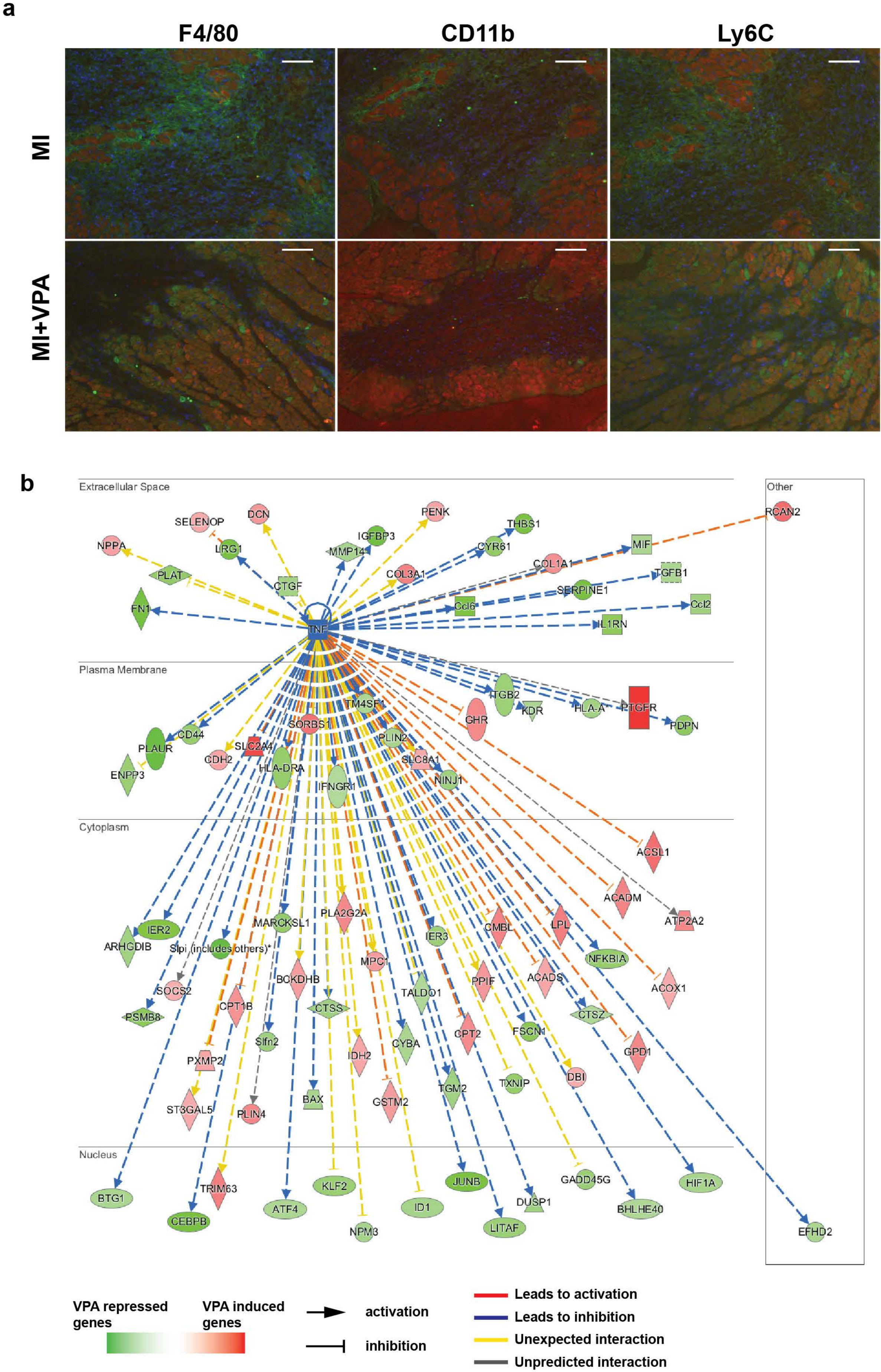
a
F4/80
CD11b
Ly6C
MI
MI+VPA
b
Extracellular Space
Other
SELENOP
DCN
PENK
THBS1
RCAN2
NPPA
LRG1
IGFBP3
MMP14
CYR61
MIF
PLAT
CTGF
COL3A1
COL1A1
TGFB1
FN1
SERPINE1
Ccl6
Ccl2
TNF
IL1RN
Plasma Membrane
ITGB2
TM4SF1
KDR
HLA-A*
PTGFR
GHR
PDPN
SORBS1
CD44
PLIN2
PLAUR
SLC2A4
CDH2
SLC8A1
ENPP3
HLA-DRA
NINJ1
IFNGR1
Cytoplasm
ACSL1
ACADM
ATP2A2
MARCKSL1
PLA2G2A
IER2
IER3
CMBL
LPL
ARHGDIB
Slpi (includes others)*
NFKBIA
MPC1
ACADS
SOCS2
BCKDHB
PPIF
ACOX1
TALDO1
CPT1B
CTSS
PSMB8
CTSZ
CPT2
FSCN1
Slfn2
CYBA
GPD1
PXMP2
IDH2
DBI
TXNIP
TGM2
BAX
GSTM2
ST3GAL5
PLIN4
Nucleus
TRIM63
KLF2
JUNB
GADD45G
HIF1A
BTG1
ATF4
ID1
DUSP1
BHLHE40
CEBPB
LITAF
NPM3
EFHD2
VPA repressed genes
VPA induced genes
activation
inhibition
Leads to activation
Leads to inhibition
Unexpected interaction
Unpredicted interaction

Figure S3

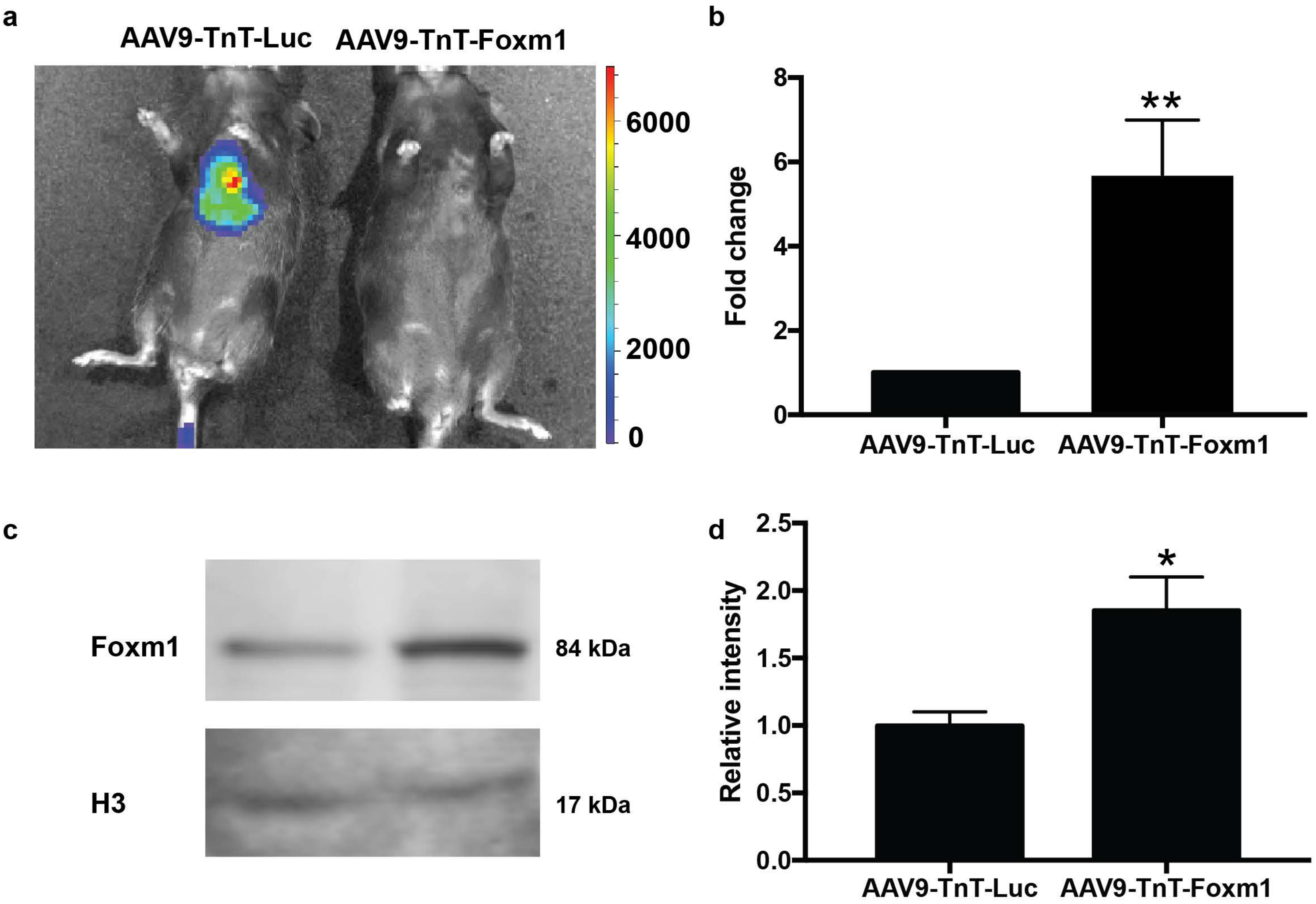

a
AAV9-TnT-Luc
AAV9-TnT-Foxm1
6000
4000
2000
0
b
Fold change
8
6
4
2
0
**
AAV9-TnT-Luc
AAV9-TnT-Foxm1
c
Foxm1
84 kDa
H3
17 kDa
d
Relative intensity
2.5
2.0
1.5
1.0
0.5
0.0
*
AAV9-TnT-Luc
AAV9-TnT-Foxm1

Figure S4

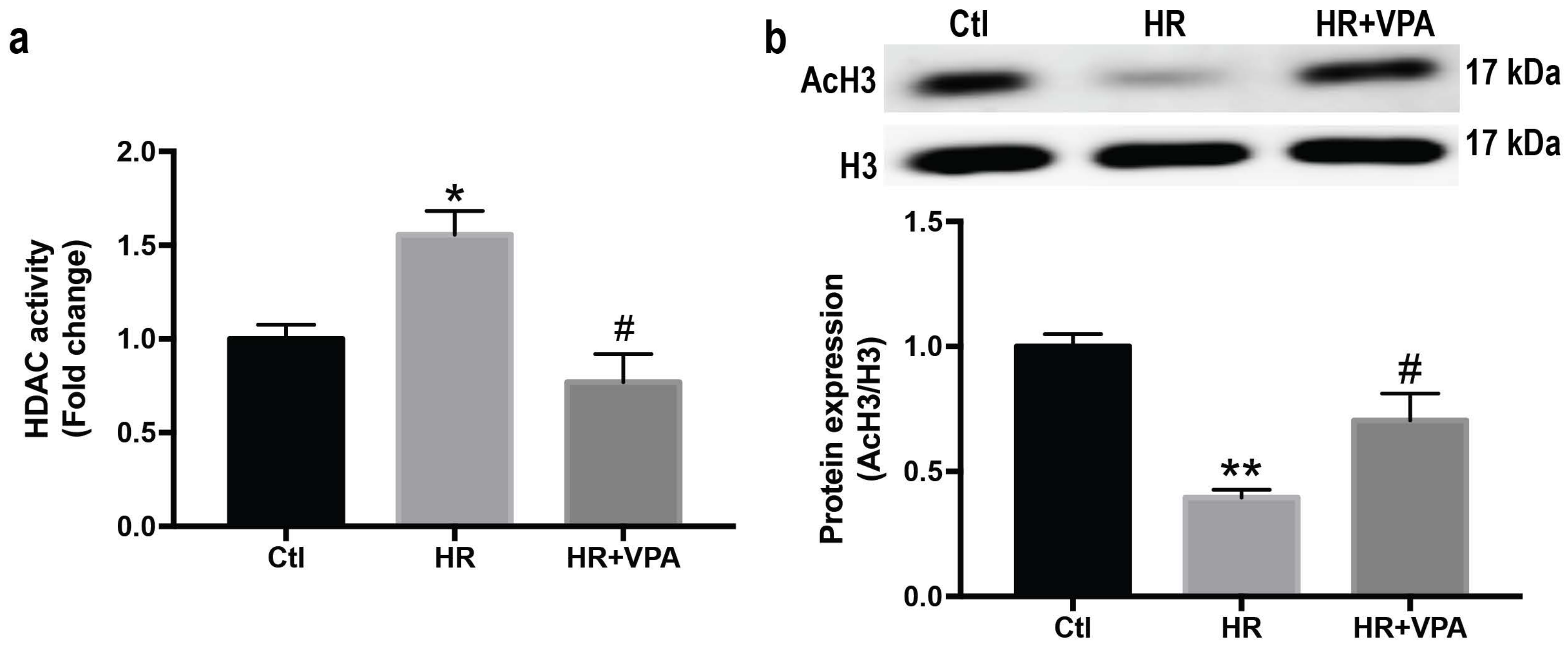
a
HDAC activity
(Fold change)
2.0
1.5
1.0
0.5
0.0
*
#
Ctl
HR
HR+VPA
b
Ctl
HR
HR+VPA
AcH3
H3
17 kDa
17 kDa
Protein expression
(AcH3/H3)
1.5
1.0
0.5
0.0
**
#
Ctl
HR
HR+VPA

Figure S5

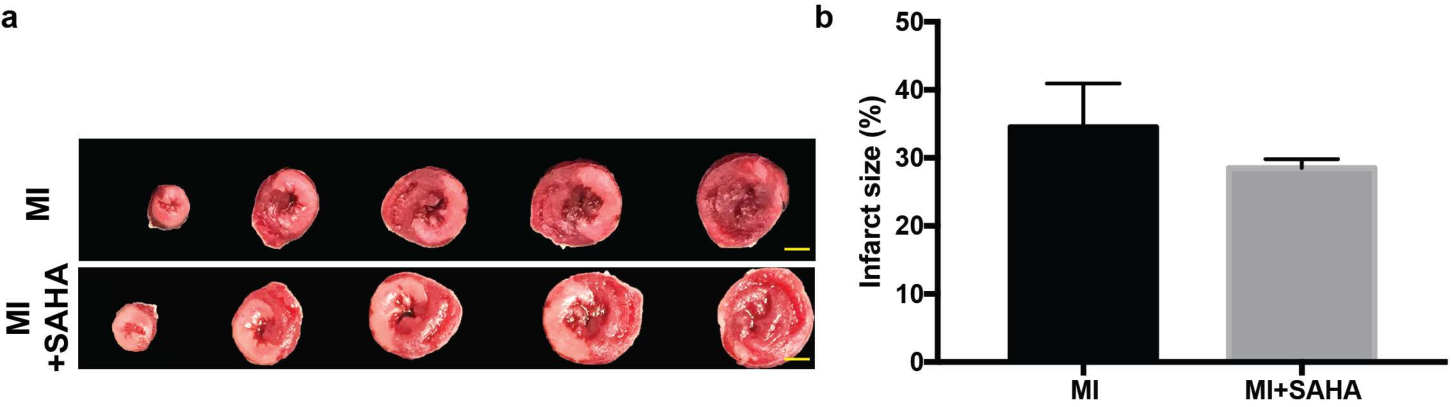

Supplement: Supplementary file 1 — Fig. S1. Volcano plot showing the effect of VPA on MI induced and repressed gene expression. Fig. S2. VPA inhibited immune response. (a) Immunostaining using F4/80, CD11b and Ly6c antibody in rats with or without VPA treatment 3 days after MI. scale bar: 25μm (b) VPA inhibited TNF network identified by Ingenuity Pathway Analysis (P < 1.0 × 10^−24^, activation z score = -4.066). (c). Treatment of VPA to neonatal rat myocytes reduced the LPS induced cell death. Fig. S3. Overexpression of Foxm1 at day 6 after injection of AAV9-TnT-Foxm1. (a) Bioluminescent image. Detection of Luciferase using AAV9-TnT virus after 6 days of virus delivery. (b) qPCR of Foxm1 expression after 6 days of injection of AAV9-TnT-Foxm1. **, P < 0.01 vs control, n = 3. (c) Representative images of Western blot of Foxm1 expression. (d) Relative intensity of Foxm1 over histone H3 from 3 replicates. *, P < 0.05 vs AAV9-TnT-Luc group, n = 3. All samples were analyzed by two tailed unpaired student’s t test. Data were expressed as mean ± SEM. Fig. S4. HDAC inhibition effects of VPA in H9C2 cells after HR. (a) HDAC activity of normal control (Ctl), HR and HR+VPA treated H9C2 cells. *, P < 0.05 vs Ctl group; #, P < 0.05 vs HR group, n = 3. (b) Representative images of western blot from control (Ctl), HR and HR+VPA treated H9C2 cells and relative intensity of histone H3 acetylation (AcH3) over histone H3 from 3 replicates. **, P < 0.01 vs Ctl group; #, P < 0.05 vs HR group, n = 3. All samples were analyzed by one way ANOVA followed by post-hoc Turkey HSD analysis. Data were expressed as mean ± SEM. Fig. S5. Effects of SAHA (125 mg/kg, SQ) on reducing infarct size at 24 h after MI in rats. (a) Representative images of heart sections by TTC staining. Scale bar: 2.5 mm (b) Quantitative analysis of infarct size expressed as percentage of left ventricle, n = 5. Data were expressed as mean ± SEM. [file mmc1.pdf]
