# Supplementary material for: HDAC inhibitor valproic acid protects heart function through Foxm1 pathway after acute myocardial infarction
Source: eBioMedicine. 2018 Dec 11;39:83–94. doi: 10.1016/j.ebiom.2018.12.003 (PMC6354709; doi:10.1016/j.ebiom.2018.12.003)
Supplement: Supplementary file 2 — Supplementary material [file mmc2.docx]

Table S1: Primers used

| Primer |  | Sequence |
| --- | --- | --- |
| Human Foxm1 | Forward | CGTCGGCCACTGATTCTCAAA |
|  | Reverse | GGCAGGGGATCTCTTAGGTTC |
| Rat Foxm1 CHIP | Forward | TTAACCGCAAGTCTGGGAC |
|  | Reverse | CTACCCACTGGGCTACCTCT |
| Mouse Foxm1 | Forward | GCAGAGGTGATCACGGAGAC |
|  | Reverse | CCAGCCCGTCAGAACTCATC |
| Mouse CYBB | Forward | GAGGTTGGTTCGGTTTTGGC |
|  | Reverse | CAGGAGCAGAGGTCAGTGTG |
| Mouse eNOS | Forward | GGGCTGGGTTTAGGGCTG |
|  | Reverse | ACTGAGGGTGTCGTAGGTGA |
| Mouse Ccnb1 | Forward | CTTGAACATGTTAGAGAAGAGAAGC |
|  | Reverse | TCGGGCTTGGAGAGGGATTA |
| Mouse Cdk1 | Forward | GGACGAGAACGGCTTGGATT |
|  | Reverse | ACACGATCTTCCCCTACGAC |
| Mouse IL-1b | Forward | TGCCACCTTTTGACAGTGATG |
|  | Reverse | TGATACTGCCTGCCTGAAGC |
| Mouse TNF-a | Forward | GGGCTTCCAGAACTCCAGG |
|  | Reverse | GCTCCTCCACTTGGTGGTTT |
| Rat Foxm1 | Forward | CTCATACCTGGTGCCCATCC |
|  | Reverse | ATAAGAGATGCTGCCAGAGG |
| Rat CYBB | Forward | TGCCAGTGTGTCGGAATCTC |
|  | Reverse | TGTGAATGGCCGTGTGAAGT |
| Rat eNOS | Forward | AAGTGGGCAGCATCACCTAC |
|  | Reverse | GCCGGCTCTGTAACTTCCTT |
| Rat Ccnb1 | Forward | CAGACGATGGTGGTGATCCA |
|  | Reverse | TCCAGTGACTTCACGACCCA |
| Rat Cdk1 | Forward | GGAACAGAGAGGGTCCGTTG |
|  | Reverse | GATCTGGCCAGTAGTCCTGTG |
| Rat IL-1b | Forward | AGGCTGACAGACCCCAAAAG |
|  | Reverse | CTCCACGGGCAAGACATAGG |
| Rat TNF-a | Forward | CTGTGCCTCAGCCTCTTCTC |
|  | Reverse | ACTGATGAGAGGGAGCCCAT |
